# Supplementary material for: Three Preceding Crops Increased the Yield of and Inhibited Clubroot Disease in Continuously Monocropped Chinese Cabbage by Regulating the Soil Properties and Rhizosphere Microbial Community
Source: Microorganisms. 2022 Apr 10;10(4):799. doi: 10.3390/microorganisms10040799 (PMC9028536; doi:10.3390/microorganisms10040799)
Supplement: Supplementary file 1 [file microorganisms-10-00799-s001.zip › microorganisms-1652351-supplementary.pdf]

Supplementary Table S1 Diversity indices of soil microbial communities based on 16S rRNA and ITS genus were analyzed from the Illumina MiSeq sequencing at a 97% sequence similarity.

**Classify**	**Treatments**	**Coverage (%)**	**No. of OTUs**	**Shannon**	**Simpson**	**Chao 1**
Bacteria	CK	98.63a	3642±79b	8.82±0.06c	0.99±0.00a	4675±167c
	B	98.80a	3982±70b	9.14±0.03b	0.99±0.00a	4995±41ab
	O	98.85a	4540±162a	9.39±0.05a	0.99±0.00a	5558±194a
	W	98.94a	4012±78b	9.12±0.06b	0.99±0.00a	4986±260ab
Fungi	CK	99.83a	408±58a	5.01±0.97a	0.88±0.07a	590±78a
	B	99.93a	218±26b	4.21±0.36a	0.87±0.03a	286±11b
	O	99.92a	231±9b	3.87±0.71a	0.81±0.10a	330±27b
	W	99.93a	270±30b	4.53±0.02a	0.88±0.01a	356±1b

Supplementary Table S2 Relative abundances of dominant bacterial phyla in soil.

	CK	B	O	W
Proteobacteria	41.051±1.202a	37.688±1.794b	35.549±1.266b	36.011±0.414b
Planctomycetes	12.449±2.739a	14.848±1.930a	15.070±0.132a	14.825±1.297a
Gemmatimonadetes	12.376±0.510ab	12.606±0.869a	10.846±0.700b	10.945±0.387ab
Acidobacteria	9.082±0.251b	9.806±1.249ab	10.949±0.309a	11.287±0.173a
Actinobacteria	10.254±1.599a	8.139±0.750a	9.696±0.562a	8.062±0.662a
Verrucomicrobia	3.748±2.103a	5.440±2.628a	7.035±2.815a	7.659±0.575a
Bacteroidetes	2.459±0.06a	3.031±0.399a	2.979±0.266a	3.106±0.458a
Chloroflexi	2.459±0.06b	2.987±0.104a	2.960±0.072a	2.918±0.025a
Firmicutes	0.873±0.06a	0.513±0.113b	0.413±0.138b	0.489±0.103b
Patescibacteria	1.847±0.474a	1.681±0.142a	1.446±0.136a	1.251±0.184a

CK represents Chinese cabbage continuous cropping, B, O, W represents bean, potato onion and wheat rotation respectively. Different letters are significantly different ($P<0.05$, Tukey's HSD test).

Supplementary Table S3 Relative abundances of dominant fungal phyla in soil.

	CK	B	O	W
Ascomycota	86.908±6.861a	87.410±3.971a	88.604±4.733a	81.693±3.819a
Basidiomycota	4.901±2.396a	2.001±1.260a	1.312±0.501a	3.412±0.737a
Mucoromycota	0.636±0.350a	0.304±0.275a	0.068±0.114a	0.244±0.284a

CK represents Chinese cabbage continuous cropping, B, O, W represents bean, potato onion and wheat rotation respectively. Different letters are significantly different ($P<0.05$, Tukey's HSD test).

Supplementary Table S4 Relative abundances of dominant bacterial classes in soil.

	CK	B	O	W
Alphaproteobacteria	25.024±0.559a	20.380±0.959b	20.172±0.692b	20.534±0.202b
Gammaproteobacteria	14.193±0.793a	14.102±0.914a	12.545±0.813a	12.604±0.151a
Gemmatimonadetes	11.435±0.532a	11.525±0.810a	9.412±0.499b	9.872±0.293b
Phycisphaerae	7.680±1.344a	8.490±0.784a	6.923±1.553a	7.760±0.999a
Planctomycetacia	4.687±1.432a	6.063±1.624a	7.955±1.536a	6.867±0.424a
Actinobacteria	6.197±1.361a	3.214±0.375c	5.283±0.431ab	3.796±0.567bc
Verrucomicrobiae	3.748±2.103a	5.440±2.628a	7.035±2.814a	7.659±0.575a
Subgroup_6	4.373±0.198a	4.498±0.609a	4.698±0.048a	4.683±0.173a
Bacteroidia	3.164±0.207a	2.943±0.381a	2.910±0.267a	3.011±0.442a
Blastocatellia_Subgroup_4	2.761±0.286c	3.130±0.440bc	3.667±0.264ab	4.006±0.275a
Deltaproteobacteria	1.825±0.188b	3.188±0.131a	2.821±0.310a	2.861±0.325a
Thermoleophilia	2.697±0.135b	3.130±0.274a	1.906±0.051c	2.430±0.086b
Acidimicrobiia	1.170±0.130c	1.577±0.116b	2.097±0.096a	1.624±0.132b
Acidobacteriia	1.101±0.127a	1.031±0.033a	1.043±0.071a	1.206±0.056a
Fimbriimonadia	0.448±0.220b	0.971±0.503ab	0.931±0.332ab	1.296±0.039a
Saccharimonadia	1.505±0.465a	0.764±0.168b	0.706±0.103b	0.523±0.047b

Supplementary Table S5 Relative abundances of dominant fungal classes in soil.

	CK	B	O	W
Sordariomycetes	57.796±20.254a	75.983±4.189a	73.551±9.394a	61.875±5.603a
Eurotiomycetes	6.397±3.902a	2.588±1.232a	3.012±0.943a	3.496±1.320a
Agaricomycetes	4.411±2.585a	1.784±1.306a	0.716±0.325a	2.776±0.651a
Leotiomycetes	9.198±6.629a	0.329±0.545a	2.159±1.153a	3.690±1.520a
Dothideomycetes	5.105±1.421a	2.723±0.406a	5.676±3.326a	5.959±3.963a

Supplementary Table S6 Mantel test analysis of soil properties and bacterial and fungal structure community of the preceding crops during harvest.

	Bacterial		Fungal	
	r	p	r	p
pH	0.4342	0.012	0.2979	0.035
EC	0.6642	0.001	0.1699	0.150
SOM	-0.02778	0.551	-0.1452	0.865
AN	0.4320	0.011	0.07141	0.368
AK	0.5469	0.001	0.3089	0.013
TN	0.007828	0.456	-0.1581	0.767
